# Observations of phase changes in monoolein during high viscous injection

**DOI:** 10.1107/S1600577522001862

**Published:** 2022-03-21

**Authors:** Daniel J. Wells, Peter Berntsen, Eugeniu Balaur, Cameron M. Kewish, Patrick Adams, Andrew Aquila, Jack Binns, Sébastien Boutet, Hayden Broomhall, Carl Caleman, Andrew Christofferson, Charlotte E. Conn, Caroline Dahlqvist, Leonie Flueckiger, Francisco Gian Roque, Tamar L. Greaves, Majid Hejazian, Mark Hunter, Marjan Hadian Jazi, H. Olof Jönsson, Sachini Kadaoluwa Pathirannahalage, Richard A. Kirian, Alex Kozlov, Ruslan P. Kurta, Hugh Marman, Derek Mendez, Andrew Morgan, Keith Nugent, Dominik Oberthuer, Harry Quiney, Juliane Reinhardt, Saumitra Saha, Jonas A. Sellberg, Raymond Sierra, Max Wiedorn, Brian Abbey, Andrew V. Martin, Connie Darmanin

**Affiliations:** a La Trobe Institute for Molecular Science, Department of Mathematical and Physical Sciences, School of Computing Engineering and Mathematical Science, La Trobe University, Bundoora, VIC 3086, Australia; b Australian Synchrotron, Australian Nuclear Science and Technology Organisation, 800 Blackburn Road, Clayton, VIC 3168, Australia; c Department of Chemistry and Physics, School of Molecular Sciences, La Trobe University, Bundoora, VIC 3086, Australia; d School of Science, RMIT University, Melbourne, VIC 3000, Australia; e SLAC National Accelerator Laboratory, 2575 Sand Hill Road, Menlo Park, CA 94025, USA; f Department of Physics and Astronomy, Uppsala University, Box 516, SE-751 20 Uppsala, Sweden; g Biomedical and X-ray Physics, Department of Applied Physics, AlbaNova University Center, KTH Royal Institute of Technology, SE-106 91 Stockholm, Sweden; h Department of Physics, Arizona State University, Tempe, AZ 85287, USA; i ARC Centre of Excellence for Advanced Molecular Imaging, The University of Melbourne, Parkville, VIC 3010, Australia; j European XFEL, Holzkoppel 4, D-22869 Schenefeld, Germany; k Research School of Physics, The Australian National University, Acton, ACT, Australia; l Center for Free-Electron Laser Science, Deutsches Elektronen-Synchrotron DESY, Notkestrasse 85, 22607 Hamburg, Germany

**Keywords:** high-viscosity injection, monoolein, lipidic cubic phase, cooling effect, continuous flow

## Abstract

Serial crystallography of membrane proteins often employs high-viscosity injectors (HVIs) to deliver micrometre-sized crystals to the X-ray beam. Typically, the carrier medium is a lipidic cubic phase (LCP) media, which can also be used to nucleate and grow the crystals. However, despite the fact that the LCP is widely used with HVIs, the potential impact of the injection process on the LCP structure has not been reported and hence is not yet well understood. The self-assembled structure of the LCP can be affected by pressure, dehydration and temperature changes, all of which occur during continuous flow injection. These changes to the LCP structure may in turn impact the results of X-ray diffraction measurements from membrane protein crystals. To investigate the influence of HVIs on the structure of the LCP we conducted a study of the phase changes in monoolein/water and monoolein/buffer mixtures during continuous flow injection, at both atmospheric pressure and under vacuum. The reservoir pressure in the HVI was tracked to determine if there is any correlation with the phase behaviour of the LCP. The results indicated that, even though the reservoir pressure underwent (at times) significant variation, this did not appear to correlate with observed phase changes in the sample stream or correspond to shifts in the LCP lattice parameter. During vacuum injection, there was a three-way coexistence of the gyroid cubic phase, diamond cubic phase and lamellar phase. During injection at atmospheric pressure, the coexistence of a cubic phase and lamellar phase in the monoolein/water mixtures was also observed. The degree to which the lamellar phase is formed was found to be strongly dependent on the co-flowing gas conditions used to stabilize the LCP stream. A combination of laboratory-based optical polarization microscopy and simulation studies was used to investigate these observations.

## Abstract 2

This is a study of the phase changes detected in monoolein samples under constant flow using a high-viscousity injector. The sample behaviour was studied using X-ray techniques while light microscopy and modelling studies were used to help interpret some of the effects observed in the data.

## 1. Introduction

Continuous sample flow injection is a key technology used to deliver protein crystals to the X-ray beam in serial crystallography experiments (DePonte *et al.*, 2008 ▸; Weierstall *et al.*, 2014 ▸). Serial crystallography aims to determine the room-temperature structure of proteins by streaming micrometre-sized protein crystals through an incident X-ray beam while collecting diffraction data (Chapman *et al.*, 2011 ▸; Boutet *et al.*, 2012 ▸). This technique is used at both X-ray free-electron lasers (XFELs) and synchrotron facilities and is an effective method for replenishing sample following interaction with the X-ray beam, which either destroys the crystal or induces radiation damage (Nogly *et al.*, 2016 ▸; Berntsen *et al.*, 2019 ▸; Hadian-Jazi *et al.*, 2021 ▸; Wiedorn *et al.*, 2018 ▸; Abbey *et al.*, 2016 ▸). Serial crystallography is also an important tool for time-resolved crystallography used to correlate structure with reaction dynamics by collecting diffraction data on millisecond-to-femtosecond timescales (Orville, 2018 ▸; Hejazian *et al.*, 2021*a* ▸). Depending on the specific requirements in terms of sample quantities and environment (Darmanin *et al.*, 2016 ▸), there are a number of different options for delivering the crystals to the X-ray beam (Hejazian *et al.*, 2021*b* ▸). The focus of the present manuscript is on high-viscosity sample injection which involves a comparatively slow-moving viscous crystal carrier being continuously streamed at a rate on the order of a few hundred micrometres per second. The slow-moving viscous stream used to inject protein crystals are often lipidic cubic phases (LCPs) which, as well as having the appropriate viscosity for injection, can also be used as a medium for growing the crystals (Cherezov, 2011 ▸). While alternative viscous media for high-viscosity injectors (HVIs) have been investigated for crystal compatibility, including grease (Berntsen *et al.*, 2019 ▸; Sugahara *et al.*, 2015 ▸, 2016 ▸; Nam, 2020*a* ▸,*b* ▸), agarose and cellulose (Sugahara *et al.*, 2017 ▸; Conrad *et al.*, 2015 ▸), LCP [typically monoolein (MO)] is the most common, particularly for membrane proteins. This is due to the compatibility of membrane protein crystals with the cubic phases of MO (Kulkarni *et al.*, 2011 ▸). HVIs also have the potential to be used to characterize other high-viscosity self-assembly materials. However, in order to extract the most useful information from HVI serial crystallography experiments and to optimize flow conditions, the impact of sample extrusion on the MO LCP structure needs to be investigated.

**Figure 1:**
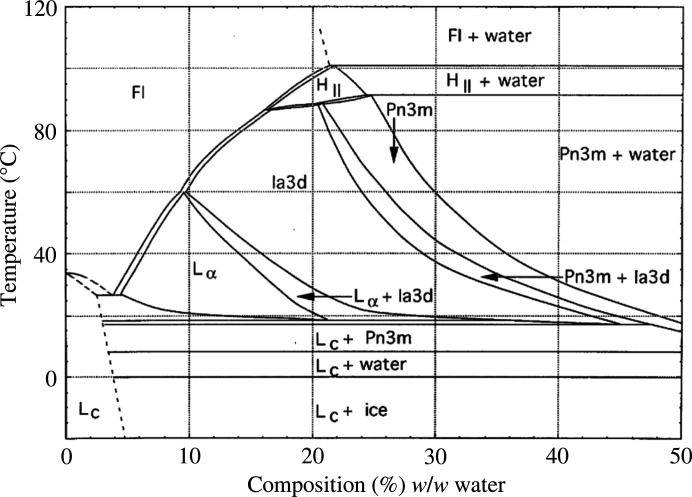
Temperature–composition phase diagram of the monoolein/water system. Reprinted with permission from Briggs *et al.* (1996 ▸), Copyright 2000 by Elsevier.

LCP is a mixture of bi-layer lipid membrane and solvent (typically water), which self-assembles into a periodic 3D structure. A key advantage of LCP is that it contains both hydro­phobic and hydro­philic components, suitable for complex macromolecules such as membrane proteins to embed and form crystals. Bacteriorhodopsin (bR) was the first membrane protein to be crystallized using MO (Landau & Rosenbusch, 1996 ▸). Therefore bR is considered a ‘standard’ for *in meso* crystallization as it crystallizes relatively easily within cubic mesophases. In previous studies the bR buffer [25 m*M* NaH_2_PO_4_ pH 5.5, 1.2% (*w*) *n*-octyl-beta-glucoside], which contained detergent, maintained the cubic phase of MO in the early stages of crystallization (Conn *et al.*, 2010 ▸). The effects of its crystallization buffer [100 m*M* citrate buffer pH 6.3, 24% (*w*) PEG2000] on MO are still unknown and need to be considered. In serial crystallography experiments the samples are transferred from syringes containing the LCP/crystals and the crystallization buffer into the injector reservoir. Although an attempt is made to remove all of the crystallization buffer, usually small amounts of it remain during injection which can affect the lipid phase. Here we focus on LCPs formed from MO in water where, depending on the lipid composition, the liquid crystalline packing can exist as lamellar, hexagonal or one of three cubic phases with zero mean curvature known as gyroid cubic, diamond cubic or primitive cubic phases. The cubic phases have the crystallographic space groups *Ia*3*d*, *Pn*3*m* and *Im*3*m*, respectively. It is these phases that exist during membrane protein crystallization, and they are assumed to exist during continuous flow in an injector. Both the phase and the lattice parameter are typically very sensitive to temperature, hydration and pressure, producing rich, complex phase diagrams (Qiu & Caffrey, 2000 ▸; Briggs *et al.*, 1996 ▸). The temperature–composition phase diagram of the MO and water system is shown in Fig. 1 ▸.

The first use of HVIs for membrane proteins involved monoolein (1-oleoyl-rac-glycerol, also referred to as mono­acyl­glycerol 9.9 or 9.9MAG) LCP as the carrier medium. However, it was discovered that a phase transition from cubic to lamellar occurred due to evaporative cooling during extrusion into vacuum (Liang *et al.*, 2015 ▸). One of the first observations of this effect was within the sample chamber at the Coherent X-ray Imaging instrument (CXI) instrument at the Linac Coherent Light Source (LCLS) (Weierstall *et al.*, 2014 ▸). This phase transition was undesirable since the X-ray beam had to be attenuated in order to prevent the sharp diffraction rings from the lamellar phase saturating (and potentially damaging) the detector. By mixing the monoolein with a small percentage of shorter-chain mono­acyl­glycerols [7.9MAG, 1-(7Z-hexadecenoyl)-rac-glycerol or 9.7MAG, 1-(9Z-hexadecenoyl)-rac-glycerol], the formation of the lamellar phase was prevented, while maintaining the integrity of the embedded protein crystals. Based on the observed transition to the crystalline lamellar phase (L_c_), the phase transition under vacuum was originally attributed to sample cooling alone without explicit consideration of the influence of the injection process.

Despite the challenges of maintaining the phase integrity of LCP during injection, owing to its biochemical and mechanical properties, it remains a key injection medium in serial crystallography experiments, particularly for membrane proteins. The effect of the lipid phase can be critical, both in terms of the LCP extrusion properties but also in terms of its inter­action with the protein crystals, for example, to avoid the collapse of ultra-swollen lipid mesophases (Zabara *et al.*, 2018 ▸) during injection. Critical to maintaining a stable and continuous LCP flow is knowledge of the rheological properties of the medium during sample injection. Although there is a significant body of literature investigating phase changes in monoolein, they are typically performed under equilibrium conditions (Briggs *et al.*, 1996 ▸; Qiu & Caffrey, 2000 ▸; Czeslik *et al.*, 1995 ▸) or highly controlled non-equilibrium conditions such as pressure jump studies (Conn *et al.*, 2008 ▸) or controlled pressure-induced phase transition studies (Pisani *et al.*, 2001 ▸). Therefore, little is known about LCP phase behaviour under continuous extrusion conditions using HVIs. An additional complication is introduced in the XFEL case in which the LCP is normally injected into a vacuum.

Here we investigate LCP phase behaviour with an HVI which incorporates a co-flowing gas stream of either nitro­gen or helium. We report on the results of small-angle X-ray scattering (SAXS) measurements of LCP under continuous flow conditions and identify the different induced LCP phases that occur. We perform experiments at both atmospheric pressure and under vacuum to compare the phase behaviour that might be expected in both synchrotron and XFEL HVI serial crystallography studies. We investigate the relationship between the LCP phase and the extrusion pressure on the sample reservoir, which is pressurized using a high-performance liquid chromatography (HPLC) pump. The X-ray scattering measurements are supported by a systematic study of the sample flow rate and gas flow using optical polarization to monitor the phase state. Due to the difference in the birefringence of the lamellar phase and the isotropic cubic phases, monitoring the optical transmission using polarized light is a well established means of determining the phase state. We also present the first simulation study of the effect of the co-flowing gas on the LCP using finite element modelling (FEM) to look at the relative effects of temperature, pressure or solvent concentration on the liquid crystal phase structure.

## 2. Methods

### 2.1. Sample preparation

Monoolein (MO, >99% purity), obtained from Sigma–Aldrich, and Milli-Q H_2_O (18.2 MΩ.cm) were used to prepare the injection medium. Where specified, water was exchanged for buffer. A standard LCP syringe mixing protocol was used to create different percentages of MO versus solvent in the gas-tight 100 µl Hamilton glass syringe coupling system [Hamilton syringes, 7656-01, Formulatrix coupler, FMLX Part 209526 (Conn *et al.*, 2010 ▸; Cheng *et al.*, 1998 ▸)]. The samples were stored in these syringes until use. Briefly, MO was added dry to one syringe barrel and in a second syringe the solvent was added. Both syringes were weighed after the addition of solvent or lipid to obtain an accurate sample solvent percentage by weight. The two syringes were coupled together, and mixing was initiated by slowly pushing the solvent into the lipid and then pushing the lipid mixture back through the coupler into the second syringe. The mixing process continued until the sample appeared homogeneous (∼20 pushes). All samples, unless otherwise stated, were prepared on the day of the experiment. For the ‘aged control’ sample (Table 1 ▸, sample C3) the mixed sample was left in a sealed syringe 6 days prior to data collection. This sample was prepared to determine whether ageing also has an influence on the lipid phase. An ‘over-mixed control’ sample (Table 1 ▸, sample C4) was also prepared to assess whether shearing forces imparted during mixing could lead to a phase change. In the preparation of this sample, the mixing in the coupler was applied more vigorously and for longer (∼50 pushes) to induce shearing forces.

Several different samples were prepared varying the solvent percentage and composition of the MO as outlined in Table 1 ▸. The sample preparation protocol was as follows:

(i) MO/water samples with compositions of 60:40 (Table 1 ▸, samples A1–3, V1, C1–4) and 85:15 (sample V2) MO/water (*w*/*v*). The percentage solvent was calculated accordingly based on the mass of MO and added to the MO.

(ii) MO in bR buffer was mixed in a 60:40 ratio MO/bR buffer (*w*/*v*) (Table 1 ▸, samples A4, V3). The bR buffer contained 25 m*M* NaH_2_PO_4_ pH 5.5, 1.2% (*w*) *n*-octyl-beta-glucoside.

(iii) MO/bR buffer + crystallization buffer (Table 1 ▸, sample A5). This sample consisted of a mixture of two components. Firstly, the MO/bR buffer component was prepared in an identical manner to the sample in (ii). This sample was then consolidated into one syringe and in the second syringe the same volume of crystallization buffer (100 m*M* citrate buffer pH 6.3, 24% PEG 2000) was added so that the final percentage of the crystallization buffer was 50%(*v*/*v*) (MO/bR buffer-to-crystallization buffer). This sample was used to mimic the process of transferring crystals from the syringe to the reservoir where the crystals are then extracted from the buffer before transfer to the reservoir. The goal was to see if any of the crystallization buffer remained in the sample and, if so, whether it affects the MO/bR buffer sample phase.

(iv) Two additional control samples, 60:40 MO/water (*w*/*v*), were made up in glass syringes as outlined above. These control samples, along with the aged and overmixed samples, were not injected through the HVI for data collection; instead the sample was extruded into a 96-well plate (Greiner bio-one microplate, half area, 675075) so that it filled the bottom of the well. The plate was then sealed with X-ray transparent plastic and placed in the X-ray beam for 10 min following extrusion into the well plates. This timescale is much longer than the expected equilibration time post-extrusion but short enough to avoid significant dehydration. This allowed any potential effects of the sample preparation protocol to be differentiated from the influence of the injection process.

### 2.2. High-viscosity injection

The LCP injector consists of a hydraulic stage, a sample reservoir and a nozzle (Weierstall *et al.*, 2014 ▸). A 40 µl reservoir was loaded with LCP matrix and connected to a fused silica capillary with either a 50 µm or a 75 µm inner diameter (Nazari *et al.*, 2020 ▸). An HPLC pump applies pressure to a plunger which forces the sample to be extruded from the capillary. The hydraulic stage multiplies the pressure applied by the HPLC pump by a factor of 14. The pressure inside the reservoir varied between 200 psi and 4000 psi, depending on the flow rate, sample composition and capillary diameter. The HPLC pump parameters were set to provide constant flow to the plunger between 0.0002 ml min^−1^ and 0.01 ml min^−1^, delivering a flow rate within the capillary that was a factor of 14 smaller than this. A co-flowing sheath of nitro­gen or helium gas was used for the synchrotron or XFEL experiments, respectively; the supply pressure was automatically adjusted in real-time between 30 psi and 300 psi to maintain a stable sample flow.

### 2.3. Synchrotron experiment

Synchrotron measurements were performed at the X-ray fluorescence microscopy (XFM) beamline at the Australian Synchrotron (Howard *et al.*, 2020 ▸) under ambient pressure in air, and at a measured temperature of 26°C. Part of the motivation behind performing these measurements at the XFM beamline was to achieve a micrometre-sized focus. The photon energy was 12.9 keV, selected by a double-crystal Si(111) monochromator. The horizontal beam was focused by Rh-coated Kirkpatrick–Baez (KB) mirrors to a spot of approximately 2 µm × 2 µm, which is smaller than the diameter of the LCP stream. The LCP injector was mounted vertically and 2D diffraction data were continuously recorded with an X-ray flux of approximately 2 × 10^10^ photons s^−1^. The detector used for data collection was an EIGER X 1M, located 800 mm from the sample, operating at a rate of 1 Hz. In addition to the HVI measurements, scattering data were also obtained from the static control samples mounted in a 96-well plate, measured under the same experimental conditions. Data from the plates were collected over an area of 1 mm × 1 mm by raster scanning the KB focused X-ray beam in a square grid pattern. Diffraction patterns were collected at intervals of 10 µm (both horizontally and vertically).

### 2.4. XFEL experiment

The XFEL experiment was performed on the CXI instrument at the Linac Coherent Light Source (LCLS) (Emma *et al.*, 2010 ▸) under vacuum conditions. The photon energy was 6 keV with a corresponding beamline transmission of around 30%; the beam focal spot size was approximately 100 nm × 100 nm. The pulse duration was 30 fs with a pulse frequency of 120 Hz. The beam was further attenuated to 1.8%, yielding an overall beamline transmission of just 0.54% transmission prior to the sample. The shot-to-shot pulse energy varied between 1 mJ and 2 mJ, corresponding to a pulse intensity of approximately 10^7^ J cm^−2^. The detector was a Cornell-SLAC Pixel Array Detector (CSPAD) (Herrmann *et al.*, 2013 ▸; Philipp *et al.*, 2011 ▸) placed 568 mm from the sample.

### 2.5. Optical polarization measurements

To investigate the effects of the sheath gas surrounding the sample stream, optical imaging with a light source and two linear crossed polarisers was performed in the laboratory. This setup was used to confirm the presence of a lamellar phase independent of the X-ray diffraction experiments. The setup consisted of an LED light source, with two linear polarisers placed both upstream (polariser) and downstream (analyser) of the LCP stream. Images were captured using a high-speed camera (iX I-speed 230 series). With this arrangement, the non-birefringent isotropic cubic phases should result in extinction (dark regions) in the image, while regions of the sample that are anisotropic (and therefore birefringent) will induce a rotation of the polarization vector resulting in some light being transmitted through the analyser. Hence, brighter regions of the transmitted image would indicate the presence of an anisotropic lamellar phase. Although by employing this method we are unable to distinguish between the specific types of LCP (*Pn*3*m*/*Ia*3*d*) or lamellar phases (L_c_/L_α_), it can provide information regarding certain phase changes with respect to sample composition, flow rate and the sheath gas pressure. Two control samples, Vaseline and silicon vacuum grease, were used as a benchmark for the optical setup. Vaseline was used as a positive control since it is known to be birefringent, whereas silicon vacuum grease was used as a negative control sample as it has no birefringence. These benchmarks confirmed that bright regions of the transmission images were associated with birefringence and not, for example, stray reflections from the sample stream.

For the optical experiments, MO/water samples were prepared with a ratio 60:40 (*w*/*w*) as described in the sample preparation section (Section 2.1). The samples were extruded through the LCP injector using a capillary with an inner diameter of 75 µm. A constant HPLC flow rate of 2 µl min^−1^ was used, which corresponds to a stream velocity of approximately 500 µm s^−1^. This setup was carried out under atmospheric pressure at 22°C. The pressure of the sheath gas was adjusted between 0 psi and 50 psi. Significant care had to be taken at very low gas pressures to prevent the sample stream from curling due to the build up of static charge.

### 2.6. X-ray data analysis

One-dimensional radial diffraction profiles were extracted from the two-dimensional X-ray diffraction patterns. Cubic phases and their corresponding lattice parameters were identified algorithmically based on the associated crystallographic space groups using a continuous wavelet transform peak-finding algorithm (Du *et al.*, 2006 ▸). To increase the signal-to-noise ratio, diffraction patterns were averaged over ten successive frames. In general, only the two peaks at the lowest scattering angle were resolvable in each pattern and, as a consequence, phase identification involved identifying ratios of the magnitudes of the scattering vectors that matched the first two peaks determined by the space group. A specific phase was determined if their ratio matched the ratio consistent with the relevant space group to within 1%. The characteristic peak ratios for the *Pn*3*m* space group are 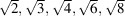 , whereas for *Ia*3*d* the ratios are 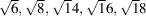 . The phases identified were cross-checked against the summed data where higher-order peaks were resolved. The matching procedure applied constraints on the lattice parameters to ensure the phases were physically realistic. This involved upper and lower bounds applied to the lattice parameters that were based on literature values (Briggs *et al.*, 1996 ▸). This helped to avoid spurious phases being identified in the scattering data, which may occur due to the matching procedure relying on just two diffraction peaks. For example, hits coming from the third and fourth peaks of the *Pn*3*m* phase, which share the same ratio as the first and second, could be eliminated. Where multiple cubic phases were identified, as was the case in much of the data captured at the LCLS, peaks often overlapped and were difficult to resolve. If this was observed, the Bonnet ratio, which specifies the ratio of the lattice parameters of two cubic phases in equilibrium, was used to further aid phase identification. The Bonnet ratio of the *Pn*3*m* lattice to the *Ia*3*d* lattice is 1.576 (Hyde *et al.*, 1984 ▸).

The presence of a crystalline lamellar phase L_c_ was indicated by the appearance of a single peak at 0.128 Å^−1^, corresponding to a *d*-spacing of 49 Å. Signal-to-noise was not sufficient to pick up higher-order peaks. The geometry of the experiment was such that the second-order peak, occurring at twice the scattering vector magnitude, would be close to the edge of the detector. The fluid lamellar phase, L_α_, manifested as a broader peak in the region 0.14 Å^−1^ to 0.175 Å^−1^, corresponding to a *d*-spacing between 36 Å and 45 Å. Compared with the crystalline peak, the L_α_ *d*-spacing was smaller and significantly more sensitive to the water concentration.

### 2.7. Gas sheath simulations

Simulations of the sample injection were performed using *COMSOL Multiphysics* (COMSOL, 2020 ▸). These models developed were previously used to simulate HVI experiments (Berntsen *et al.*, 2022 ▸) and were updated for the present study by introducing an additional component consisting of the surrounding sheath gas. The simulations used a 75 µm-diameter capillary to match the optical polarization experiments. To model the gas flow, the gas velocity was slowly increased from zero up to 100 m s^−1^. The ramping was slow enough such that the system was in a steady state at each gas velocity. Temperature, pressure and velocity profiles of the gas stream were then extracted at different velocities. Relative to the gas speed the sample stream flow was negligible and for simplicity was set to zero in the FEM simulations.

## 3. Results and discussion

### 3.1. Phase compositions of monoolein/water systems observed in air at the synchrotron

The phases and descriptions of all samples are presented in Table 1 ▸. Of the three MO/water samples (Table 1 ▸, samples A1–3), two exhibited a cubic phase, consistent with the expected results based on the temperature–solvent composition studies investigated by Briggs *et al.* (1996 ▸). These samples also exhibited a coexisting additional lamellar phase (Table 1 ▸, samples A1–2).

#### 3.1.1. Cubic phases observed in MO/water in air

Firstly, to understand the cubic phase behaviour, a lipid:water ratio of 60:40 at 26°C sits on the phase boundary between the *Pn*3*m* and *Ia*3*d* phases, where a small increase/decrease in temperature and/or solvent content can result in the sample phase being in either the *Pn*3*m* or the *Ia*3*d* phase or both. Briggs *et al.* (1996 ▸) measured a lattice parameter of around 102 Å for the *Pn*3*m* phase under these conditions, which is consistent with our results for sample A1 (Table 1 ▸). At various times sample A3 exhibited both the *Pn*3*m* and the *Ia*3*d* phases, although the phases were not observed to co-exist simultaneously within the sample stream. The co-existence of the *Ia*3*d* and *Pn*3*m* is as predicted by the Bonnet ratio.

Sample A2 was the only MO/water sample to exhibit no cubic phases that corresponded to equilibrium behaviour. This sample exhibited an *Ia*3*d* phase with a lattice parameter of 136 Å, significantly smaller than expected for this sample composition (Briggs *et al.*, 1996 ▸). We attribute this to dehydration of the sample, since other factors that may influence the lattice parameter – evaporative cooling or a pressure-induced change inside the injector – have been shown to result in an increased lattice parameter (Czeslik *et al.*, 1995 ▸). The control samples C1 and C2 also showed a reduced lattice parameter, suggesting dehydration cannot be attributed to the injection process. This suggests that water loss during mixing or sample loading, rather than injection, accounts for the reduced lattice parameter detected in sample A2.

Sample A3 also showed *Pn*3*m* and *Ia*3*d* phases with lattice parameters close to the expected equilibrium values. However, for a short time near the start of data collection it exhibited a *Pn*3*m* phase with a lattice parameter of 91 Å, smaller than the expected equilibrium value at hutch temperature (26°C). We note this observation as an effect of the non-equilibrium nature of the injection process.

**Figure 2:**
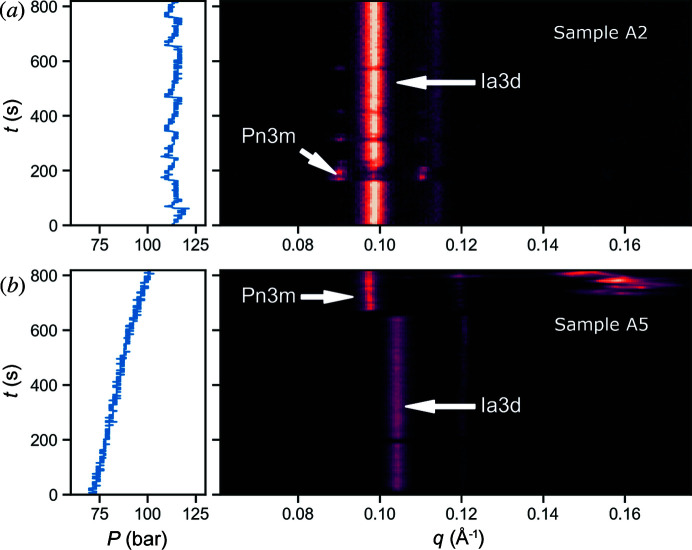
Illustrative data showing phase changes between the *Ia*3*d* and *Pn*3*m* cubic phases for samples injected in air at the Australian Synchrotron: (*a*) sample A3–MO, water 60:40 (*w*/*w*); (*b*) sample A5–MO/bR buffer and crystallization buffer 50:50. Reservoir pressure is shown in the left-hand plots, and radial scattering profiles are shown in the right-hand plots.

The phase changes observed for sample A3 appeared almost instantaneously, which is apparent from the time dependence for the illustrative sample run shown in Fig. 2 ▸(*a*). The *Ia*3*d* peak is dominant in the sample run and the *Pn*3*m* peaks only appear occasionally. The sample composition is near the phase boundary between *Pn*3*m* and *Ia*3*d*, and the phase behaviour may be sensitive to small changes in the local environmental conditions. The phase boundary has a co-existence region, and the sudden change of phase is difficult to account for as a dynamical transition. Pressure effects are known to cause phase changes (Berntsen *et al.*, 2022 ▸), but we have not observed any correlation between the reservoir pressure (shown in the left-hand panels of Fig. 2 ▸) and the phase behaviour. Dehydration and/or cooling effects due to evaporative cooling induced by the sheath gas may also play a role.

However, all the MO/water samples were prepared in the same way and showed some degree of inherent sample inhomogeneity in relation to its water content which can account for the observed variations in the cubic phase/lattice structure. However, what remains constant is the cubic phase is maintained in all samples and the lamellar phase could not be reproduced in the control plate samples (C1–C4) even if the sample was over-mixed.

#### 3.1.2. Crystalline lamellar phase observation in synchrotron data

**Figure 3:**
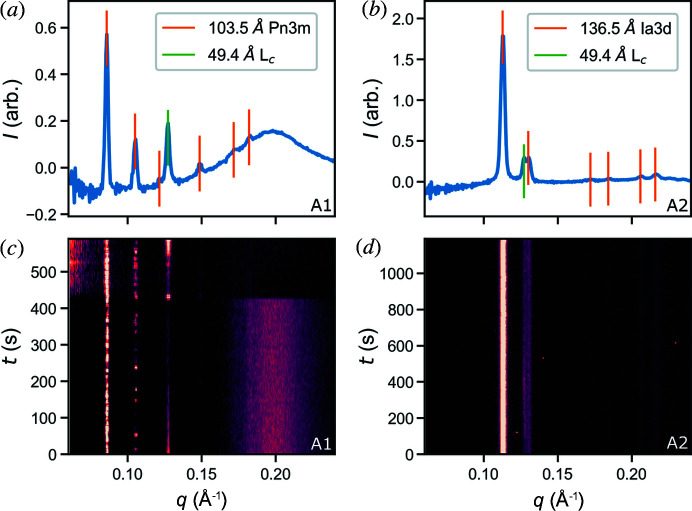
Radial diffraction profiles showing the coexistence of crystal lamellar (L_c_) and cubic (*Pn*3*m* and *Ia*3*d*) phases when injected into air at the Australian Synchrotron: (*a*, *c*) sample A1, (*b*, *d*) sample A2. Both samples were mixed with the ratio of 60:40 MO/water (*w*/*w*). Panels (*a*) and (*b*) represent the average diffraction profile. Vertical lines indicate the peak positions implied by the crystallographic space group and the stated lattice parameter. Panels (*c*) and (*d*) show the evolution of the diffraction profiles over the course of the data collection of a single sample and indicate the simultaneous presence of the lamellar and cubic phases.

We now address the unusual behaviour of the coexisting lamellar phase in the 60:40 ratio MO/water samples. An unexpected feature of the diffraction profiles was the presence of a lamellar peak, coexisting with cubic phases, in the majority of two of the water samples tested. In samples A1 and A2 (Table 1 ▸), a lattice spacing of 49 Å (peak at 0.128 Å^−1^) was detected (Fig. 3 ▸). For these samples in which both the L_c_ phase and a cubic phase appear, they are generally observed concurrently. This is shown in Figs. 3 ▸(*c*) and 3(*d*), where the persistence of each of these phases can be observed. Based on published literature this is identified as the primary peak of the crystalline lamellar phase L_c_ (Briggs *et al.*, 1996 ▸). According to phase diagrams the L_c_ phase is normally observed at temperatures below 18°C (Qiu & Caffrey, 2000 ▸). Though wide-angle X-ray scattering (WAXS) is typically required to definitively distinguish the crystalline lamellar L_c_ phase from the fluid lamellar L_α_ phase, we note that the measured *d*-spacing of 49 Å is typical of the L_c_ phase and is larger than the spacing observed for the L_α_ phase (which ranges from 40 Å to 45 Å). Furthermore, the observed peak is sharp and appears at a consistent scattering angle across several data runs and therefore we can assume this is an L_c_ peak. MO/water solutions are also known to form inverse hexagonal phases that produce diffraction peaks at similar angles; however, these occur only at 90°C or above (Briggs *et al.*, 1996 ▸) or with the addition of salts or other additives (Borné *et al.*, 2001 ▸) and therefore can be ruled out.

Coexistence of a cubic and lamellar phase under the experimental conditions used here (injection into air at ambient pressure) has not been reported previously in the literature. A metastability study (Qiu & Caffrey, 2000 ▸) found these phases may exist in equilibrium at temperatures below 18°C. They reported that, at temperatures between 17°C and 18°C, *Ia*3*d* and L_c_ coexist in equilibrium, while between 8°C and 17°C, *Pn*3*m* and L_c_ coexist. That study was performed under strict sample preparation conditions to avoid inducing metastable phases. Earlier work by Briggs *et al.* (1996 ▸), which did not attempt to avoid metastable cubic phases, also did not observe L_c_ at water concentrations above 15.2% at any temperature; temperatures between 0°C and 100°C were surveyed in that study. In the present synchrotron experiment, since the data collection temperature was maintained at 26°C in the hutch, it is not expected that the environmental temperature would be sufficient to induce this phase change.

The data from our control samples, C1 and C2 (Table 1 ▸), show no sign of a lamellar peak. This indicates that the lamellar phase was not created during the sample preparation process, but rather is associated with the injection process.

#### 3.1.3. Phase compositions of monoolein/buffer systems during injection

The addition of complex buffers to MO is known to produce complex LCP behaviours. The addition of detergents, salts and other additives can change the phase of MO without altering the solvent percentage (Misquitta & Caffrey, 2003 ▸; Conn, Darmanin, Mulet, Le Cann *et al.*, 2012 ▸). To identify how buffers impact the sample behaviour during injection, data from two buffer systems were analysed (Table 1 ▸, samples A4–5). Sample A4, with a ratio of 60:40 MO/bR buffer, was observed to have a stable *Ia*3*d* phase as expected (lattice parameter of 149 Å), as well as a lamellar phase (49 Å). Previous investigations performed in a plate system also revealed the existence of the *Ia*3*d* lattice but with a slightly higher lattice parameter (161.5 Å); however, no lamellar peak was identified (Conn *et al.*, 2010 ▸). Therefore, the existence of the lamellar and smaller *Ia*3*d* lattice parameter is linked to the continuous flow of this sample.

Sample A5 contained a more complex buffer mixture compared with A4, with the addition of a crystallization buffer to an existing LCP mixture. This resulted in the presence of both the *Pn*3*m* and the *Ia*3*d* phases in the sample. This sample has excess solvent which is expected to lead to a predominant *Pn*3*m* phase. However, the behaviour of this sample was inconsistent, with other transient peaks appearing at low scattering angles that could not be assigned to any phase. These could be attributed to the additives contained within the crystallization buffer. Similar to MO/water (sample A3), the two cubic phases in sample A5 did not coexist in the same image, instead the phase jumped from *Pn*3*m* to *Ia*3*d* [Fig. 2 ▸(*b*)]. It is likely that the sample composition contributes to these effects, as a high concentration of salts and additives can induce a phase change or maintain certain phases within the sample over time (Conn, Darmanin, Mulet, Hawley *et al.*, 2012 ▸; Conn, Darmanin, Mulet, Le Cann *et al.*, 2012 ▸).

### 3.2. Comparison of air injection at the synchrotron with vacuum injection at the XFEL

#### 3.2.1. Observation of a L_α_ phase vacuum data

**Figure 4:**
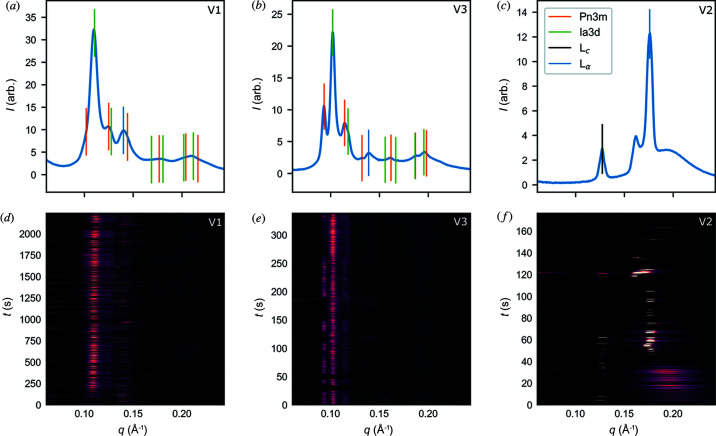
Radial diffraction profiles captured during injection into vacuum at the LCLS. The two samples prepared with a 60:40 MO/water ratio, (*a*, *d*) V1 and (*b*, *e*) V3, exhibit the *Ia*3*d* and *Pn*3*m* phases occurring simultaneously. The positions of peaks associated with these phases are shown as vertical lines in plots (*a*)–(*c*). An L_α_ peak is also observed in these samples with a structure parameter of around 45 Å. The 85:15 MO/water sample, (*c*, *f*) V2, was the only sample observed with an L_c_ phase during vacuum injection. The L_α_ phase with the structure parameter 37.5 Å is also observed in this data, which is consistent with the previous studies for this composition.

X-ray scattering data were collected under vacuum at the CXI beamline at the LCLS on identical samples to those measured in air. Several differences are evident between the air injection results at the Australian Synchrotron and the vacuum results at the LCLS (Table 1 ▸, Fig. 4 ▸). The vacuum data show co-existence of *Pn*3*m* and *Ia*3*d* phases, which was not observed in air. However, co-existence of these phases is reported in the phase diagram and may be due to a reduction in water content from the initial composition.

One observation common to both the vacuum and the air data collection is the coexistence of a lamellar phase, observed in a 60:40 ratio for both MO/water and MO/bR buffer samples. Surprisingly it was the L_α_ phase observed during injection under vacuum instead of the L_c_ phase. Transient peaks were observed in the range 0.14–0.15 Å^−1^ for both MO/water and MO/buffer samples, which is consistent with the primary peak position of an L_α_ phase. The L_α_ peak in the vacuum data was transient, broader and did not have a consistent scattering angle, in contrast to the L_c_ peak that appeared sharp and persisted for long periods of time in the air data. This observation is supported by the published data, as the L_α_ phase has been detected between 38 Å and 48 Å at temperatures similar to those investigated here (Briggs *et al.*, 1996 ▸).

#### 3.2.2. Observation of co-existent L_c_ and L_α_ phases

To confirm the presence of the L_α_ phase, a control L_α_ phase sample was prepared based on the MO/water composition phase diagram (Briggs *et al.*, 1996 ▸). With an MO/water ratio of 85:15 it is expected the phase should be predominantly L_α_. Analysis of the data from this sample collected under vacuum showed the expected L_α_ phase was altered during the injection process. Our analysis showed the presence of both L_α_ and L_c_ phases, which has not been observed previously in HVI experiments [Table 1 ▸; Figs. 4 ▸(*c*) and 4(*f*)]. A possible explanation is that, at only 15% water content, this sample requires minimal cooling and/or dehydration to induce a transition to the L_c_ phase (Qiu & Caffrey, 2000 ▸). This could occur at the surface of the sample stream while the internal part of the sample retains the original level of hydration. Although this does not answer the question of why we see the L_α_ phase in 60:40 ratio samples it does provide an indication of the water movement/loss in the sample stream to the environment, which needs to be considered when designing HVI experiments.

### 3.3. Comparison of lattice parameters between air and vacuum data

The observed lattice parameters of cubic phases in the MO/water samples were reduced under vacuum compared with those in air (see Fig. 4 ▸). For the V1 sample, 60:40 MO/water, the *Pn*3*m* and *Ia*3*d* parameters were 87 Å and 138 Å, respectively. In the phase diagram for MO (Briggs *et al.*, 1996 ▸) at ambient pressure lattice parameters decrease with temperature, which is the opposite to the cooling effect expected during vacuum injection. Hence, temperature does not explain the reduced lattice parameters. Dehydration after injection could potentially explain the discrepancy, although equilibrium studies suggest the *Pn*3*m* phase would vanish if this were the case (Briggs *et al.*, 1996 ▸). Lower pressure can result in increased curvature of the lipid bilayer, and hence a smaller lattice parameter (Czeslik *et al.*, 1995 ▸), which may be a plausible explanation for these observations.

Interestingly, the reduced lattice parameter was not observed for the MO/buffer mixture, sample V3, for which the *Ia*3*d* parameter was comparable with that for air injection. This may be a result of the buffer components ameliorating the effects of low-pressure or resisting dehydration of the sample.

### 3.4. Pressure effects in sample reservoir

**Figure 5:**
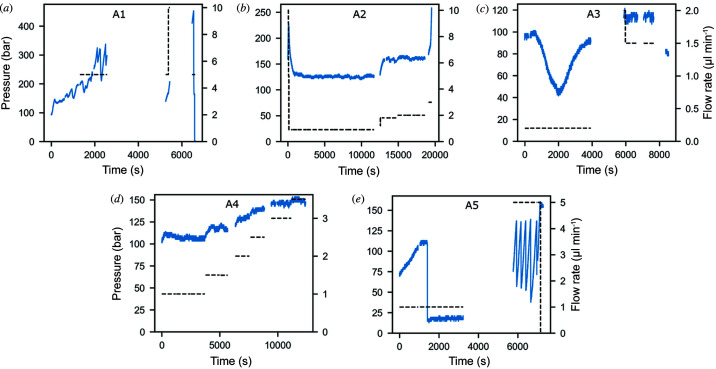
Variation of pressure and flow rate during the synchrotron experiment. Pressure is depicted by the solid blue line; the dashed black line is the flow rate. Breaks in the plot correspond to periods where data were not collected. Flow rate was adjusted manually through the HPLC pump in order to maintain a steady flow. The true flow rate of the sample leaving the nozzle may be different, particularly when the ‘saw-toothing’ behaviour occurs. In general, the true flow rate also lags a change in the HPLC set point as the sample takes some time to adapt. Plots (*a*)–(*f*) depict data for samples A1–A5, respectively. See Table 1 ▸ for sample composition.

Pressure plays an important role in driving phase changes in MO (Pisani *et al.*, 2001 ▸; Conn *et al.*, 2008 ▸; Czeslik *et al.*, 1995 ▸). Given the design of the HVI and how the sample is extruded, changes in the pressure inside the sample reservoir could potentially induce phase changes in the lipid sample. The sample injection uses a plunger, attached to a water line, connected to an HPLC pump. The plunger is placed at the top of the sample and the water flow rate is adjusted at the HPLC pump which then initiates the movement of the plunger causing the sample to be extruded out of the reservoir. Through the HPLC we can record the pressure applied to the plunger to generate the desired sample flow rate. The reservoir pressures varied greatly across the experiment from ∼200 psi to 4000 psi. Fig. 5 ▸ shows the time-dependence of the reservoir pressure (solid line) as well as the flow rate set point of the HPLC pump (dashed line). We observed a significant lag between changes in the HPLC flow rate and the response in sample flow, as the system took time to reach equilibrium which needs to be considered when interpreting pressure effects in the stream.

We observed substantial variation in pressure for the same sample within a single run, at times the pressure would evolve in a ‘saw-tooth’ pattern, gradually building before dropping again rapidly. This behaviour is best illustrated by sample A5 [Fig. 5 ▸ (*e*)]. However, no direct correlation between reservoir pressure and sample phase was observed. Interestingly, these rapid jumps in reservoir pressure that occurred spontaneously did not align with any significant observed change in the sample phase. Similarly, changes in phase, such as those depicted in Fig. 2 ▸, were not accompanied by noticeable pressure changes. This is shown in the left-hand panels of Fig. 2 ▸, which depict the pressure variation. Therefore, it seems the change in pressure associated with driving the plunger in the reservoir does not have a significant impact on the phase of the lipid in the stream.

The published data in the literature corroborate the conclusion that reservoir pressure observed in the injector is insufficient to cause a phase change (Pisani *et al.*, 2001 ▸; Conn *et al.*, 2008 ▸). Studies investigating pressure dependence of the MO/water system have previously identified a transition from the *Pn*3*m* to lamellar phase at 49 Å occurring at pressures greater than 5800 psi (Pisani *et al.*, 2001 ▸). Other published pressure jump studies (Conn *et al.*, 2008 ▸) show that a transition from *Pn*3*m* to *Ia*3*d* can also be triggered in lipids by a sudden jump to very high pressures, exceeding 15000 psi. However, the pressures used in the present study do not reach these threshold values (typical pressures are around 2000 psi). Extrapolating from these results, it seems reasonable to conclude that the pressure extrusion of the lipid through an HVI capillary is unlikely to be associated with the observations of *Ia*3*d* and lamellar phases.

### 3.5. The effect of the sheath gas on sample flow

The role of the sheath gas is to stabilize the sample stream (DePonte *et al.*, 2008 ▸; Nogly *et al.*, 2016 ▸; Weierstall *et al.*, 2014 ▸). The sheath gas around the sample stream forces the sample to move away from the nozzle tip and stream downwards, creating a continuous flow. However, if the gas flow is too high it will destabilize the stream and cause discontinuities in the jet. Therefore, there is a need to optimize the gas flow for each measurement, and this value depends on the nozzle tip used, type of sample and how the nozzle is assembled. During our optical experiments a stable sample flow could be reliably achieved when the backing pressure of the sheath gas was set between 20 psi and 30 psi. Above approximately 40 psi, the gas flow was sufficient to cut the stream close to the nozzle tip. Below 20 psi the sample stream tended to curl up towards the nozzle tip, likely due to the build up of static charges. It was discovered that if we started with a steady stream and gradually decreased the gas flow, a stable stream with sheath gas flow of zero could be achieved for short periods of time.

**Figure 6:**
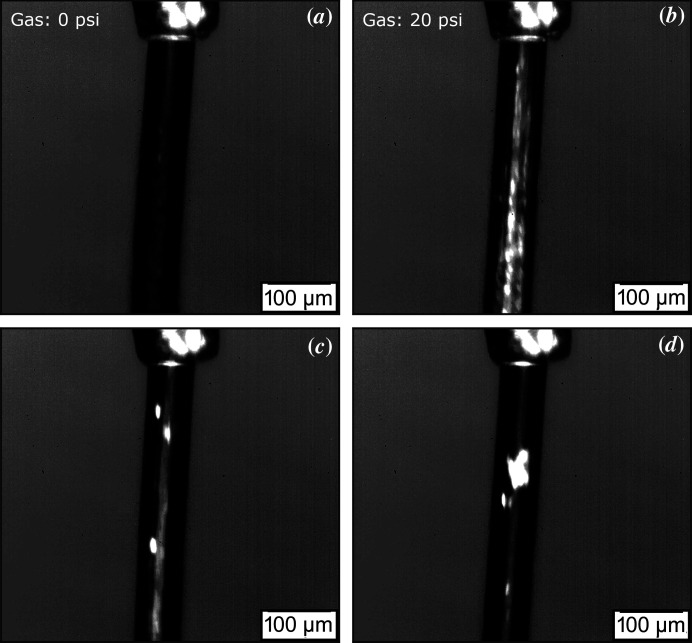
Images of the sample stream taken in air with the sample between crossed polarisers. Bright regions of the images indicate the presence of the polarizing lamellar phase. The sample composition is 60:40 MO/water (*w*/*v*) and the nozzle diameter is 75 µm. (*a*) Stabilizing gas sheath turned off and sample is in a non-polarizing cubic phase. (*b*) Gas sheath on with backing pressure of approximately 20 psi. Brightness of the sample indicates the presence of lamellar phase. (*c*, *d*) The appearance of isolated lamellar regions not associated with changes in gas pressure. These are likely caused by air bubbles within the sample and are consistent with the transient appearance of L_α_ observed in the X-ray data.

To study the effects of the sheath gas flow, optical polarization experiments were performed. This method has been used for decades to identify lipid mesophases (Lee & Kellaway, 2005 ▸). An isotropic material, such as LCP, has the same refractive index in all directions and interacts with polarized light the same in every direction (Smallman & Ashbee, 2013 ▸). By comparison, anisotropic materials such as the lamellar or hexagonal lipid phases (Bubnov *et al.*, 2013 ▸) have different refractive indexes in different directions that interact with polarized light (Smallman & Ashbee, 2013 ▸). Therefore, the LCP appears as a dark background under a polarizing microscope, whereas other lipid phases will yield a characteristic interference pattern, which is indicative of birefringence (Bubnov *et al.*, 2013 ▸; Lee & Kellaway, 2005 ▸). We assume that any birefringence observed in the stream is due to the presence of a lamellar phase, because this phase was observed in the X-ray data. Figs. 6 ▸(*a*) and 6 ▸(*b*) show the effect of the gas sheath on the polarization state of the sample stream. The presence of a lamellar phase can be inferred from the appearance of bright regions in panels (*b*), (*c*) and (*d*). Sample regions where the lamellar phase was observed could be classified into two distinct categories: one in which small discrete pockets travel within the stream, starting prior to the sample leaving the nozzle, as shown in Figs. 6 ▸(*c*) and 6(*d*). These are likely due to air bubbles arising within the sample where the air–lipid interface leads to local drying effects within the sample and the formation of an L_α_ phase. This is consistent with the transient observations of an L_α_ phase in the X-ray data and there does not appear to be any correlation between their occurrence and experimental parameters such as gas backing pressure, sample flow rate or reservoir pressure. The second category is where substantial regions of the sample exhibit birefringence simultaneously and more consistently. The degree to which the sample is polarized depends on the distance from the nozzle rather than flowing with the stream which suggests that the sample may dehydrate the further away from the nozzle it travels (*i.e.* solvent loss to the gas sheath) or it could possibly be a flow effect (*i.e.* due to cooling). This is depicted in Fig. 6 ▸(*b*). This latter case is assumed to be the L_c_ phase that is observed which is supported by the X-ray data.

The appearance of a lamellar phase was strongly correlated with the gas flow. When the co-flowing gas was absent the sample remained in an isotropic state, attributed to the cubic phase [Fig. 6 ▸(*a*)]. This suggests that the co-flow gas does induce the change to a lamellar phase. However, the converse was not always true and occasionally the stream did not exhibit birefringence regardless of the presence of the sheath gas. Four samples were prepared with the 60:40 MO/water composition to test this hypothesis and we conclude from the measurements that the sheath gas can induce birefringent behaviour in the sample stream but not consistently. All the samples that were tested exhibited birefringence at various stages of extrusion from the reservoir, suggesting some degree of sample inhomogeneity is a common feature of the injection process. The lack of consistent polarization agrees with the X-ray data, for which the L_c_ phase was persistent but not universally present.

**Figure 7:**
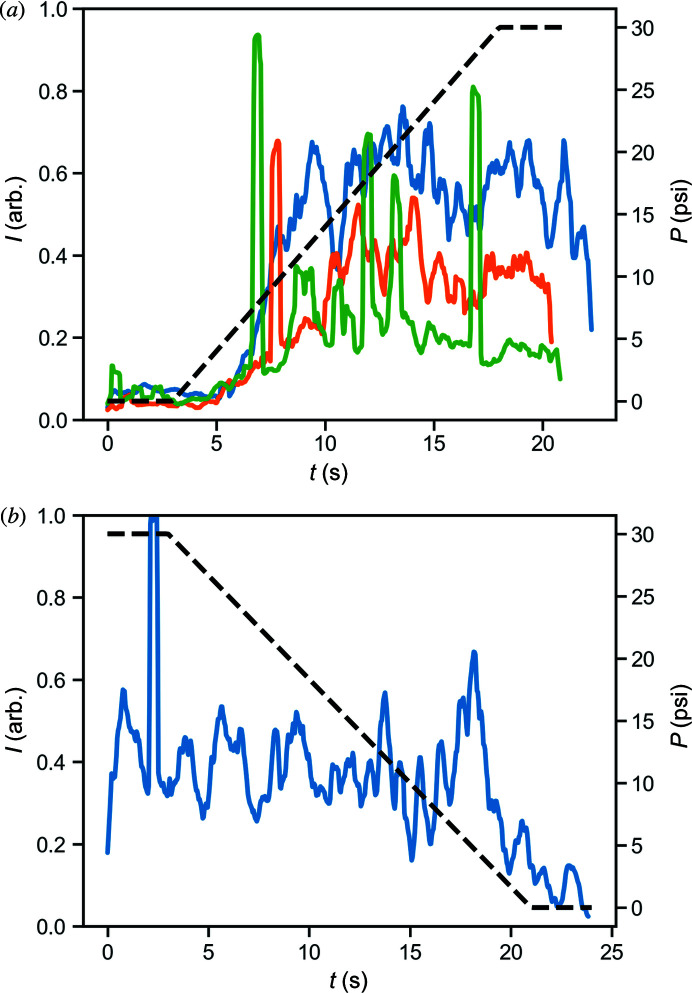
Intensity of light transmitted through the sample stream 100 µm from the nozzle tip as a function of time, as the stabilizing gas flow is varied. Black dashed line shows the approximate backing pressure of the gas jet. High transmission is indicative of the presence of lamellar phase while low transmission indicates the sample is in a cubic phase. The sample composition is 60:40 MO/water (*w*/*w*). (*a*) Gas backing pressure is reduced slowly to zero prior to collecting data, then increased steadily from 0 psi to 30 psi. A significant increase in intensity is observed, indicating a transition to the lamellar phase. This was repeated three times to show the procedure was reproducible. (*b*) Gas backing pressure begins at 30 psi and is reduced steadily to zero. The corresponding decrease in transmission indicates reversion to LCP as the gas flow is reduced.

The backing pressure of the sheath gas appears to be a key variable in determining the degree to which the lamellar phase appears. The effect of backing pressure was studied by reducing the pressure to zero, then increasing steadily to 30 psi. The reversibility of this process was demonstrated by beginning at 30 psi and reducing gas flow to zero. The presence of the lamellar phase at each point in time was quantified by integrating the brightness of the image over a region 100 µm from the nozzle tip, corresponding to the point at which X-ray data were collected while the gas backing pressure was adjusted. The results shown in Fig. 7 ▸(*a*) demonstrate the effect of increasing the gas backing pressure from zero to 30 psi, while Fig. 7 ▸(*b*) illustrates the effect of decreasing gas pressure to zero. The sharp peaks in intensity found in both plots correspond to L_α_ regions passing through the interaction region. These results clearly show the co-flowing sheath gas plays a significant role in causing a transition in the sample from the LCP to the lamellar phase. This effect appears to ramp up quickly when the gas jet is turned on, then plateaus as a function of gas backing pressure after the initial ramp-up. Our observations thus far indicate the gas flow rate is important, not only for stability of the stream but also for the phases that are present in MO which can contribute to the lamellar phase we observe in our data. However, temperature is another key parameter which needs to be considered carefully. Although the experimental temperature remains constant the gas stream may act to cool the surface temperature of the HVI stream. To investigate this, temperature changes due to gas flow were modelled using FEM simulations.

### 3.6. Simulations of the effect of the sheath gas

Finite-element modelling (FEM) simulations were performed to understand the effect of the sheath gas on the LCP stream and assist the interpretation of experimental data. The temperature, pressure and velocity profiles of the gas sheath were calculated at various gas flow speeds. The simulations do not account for evaporative cooling as water leaves the system or water loss to the environment.

**Figure 8:**
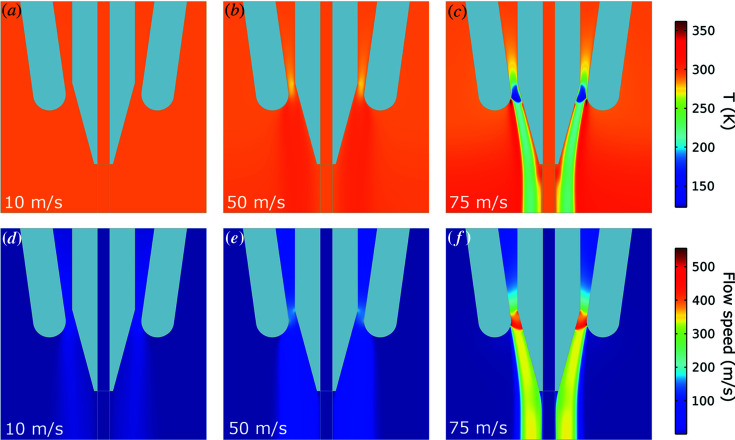
(*a*)–(*c*) Simulated temperature and (*d*)–(*f*) velocity profiles of a nitro­gen sheath gas surrounding a capillary with a 75 µm internal diameter. Gas flow speed is 10 m s^−1^ in (*a*) and (*d*); 50 m s^−1^ in (*b*) and (*e*); and 75 m s^−1^ for (*c*) and (*f*).

The temperature and velocity results are shown in Fig. 8 ▸. At low gas-flow speeds (10 m s^−1^), the gas temperature is not significantly different from the ambient gas temperature surrounding the sample stream. At higher speeds (50 m s^−1^), the gas temperature becomes somewhat warmer than the surrounding air. As the speed is increased further (75 m s^−1^), the temperature profile changes dramatically, and the gas jet becomes significantly colder. The temperature profile [Fig. 8 ▸(*c*)] indicates that the temperature can drop from 26°C down to just 1.85°C at the gas/sample interface. Based on the Qiu & Caffrey (2000 ▸) phase diagram, at such low temperature coexistence of cubic phase and L_c_ phase is possible. Although we cannot measure the sheath gas speed around the extruded sample directly using our experimental setup, estimates of sheath gas flow based on the backing pressure and size of the gas aperture indicate we are in the correct flow regime. This suggests the gas stream may induce cooling of the sample surface which in turn could account for the observed L_c_ phase in our X-ray data.

## 4. Conclusions

We have reported on the liquid crystal phase composition of lipidic systems during high-viscosity injection. We found that the injection process can induce a partial phase change of the lipid stream resulting in the coexistence of cubic and lamellar phases. Interestingly, when the HVI was placed under vacuum it induced an L_α_ phase, while in air the L_c_ phase was present. Optical polarization experiments revealed that this transition was highly sensitive to the presence and conditions of the sheath gas, which is often used to stabilize the lipid stream in HVI experiments. The temperature/concentration phase diagram for the MO/water system indicates that the L_c_ phase is stable at temperatures below 17°C across a range of solvent concentrations, suggesting a cooling effect is likely to be a key driver of the transition to an L_c_ phase in air; we note that this is also supported by our gas-flow simulation results. However, the effects of dehydration at the surface of the sample stream as well as variations in the permeability of the sample, which were not included in the simulation, may also play a role in driving lipid phase changes. The observation of the L_α_ phase as opposed to the L_c_ phase during vacuum injection is interesting given that one would expect cooling effects to be exacerbated by injection into vacuum. Overall, these observations confirm the impact of sample injection on the phase state of LCP which is commonly used as a sample carrier for serial crystallography experiments. A deeper understanding of the behaviour of the lipidic material under these conditions is imperative, particularly if HVI applications beyond just serial crystallography, including within soft materials, are to be explored.

## Figures and Tables

**Table 1:** Phases and corresponding lattice parameters calculated from X-ray diffraction profiles obtained during HVI of monoolein samples Samples injected in air at the synchrotron and under vacuum at the XFEL are denoted A and V, respectively. The injector nozzle diameter was 75 µm unless otherwise stated. Control samples, denoted C, were deposited in a 96-well plate for lattice parameter determination without injection. Where multiple phases appear on the same row, the phases were observed concurrently in the diffraction data.

Sample	Composition	*Pn*3*m* (Å)	*Ia*3*d* (Å)	Lamellar (Å)
Synchrotron (air)				
A1	MO/water 60:40, 50 µm nozzle	103		49
A2	MO/water 60:40		136	49
A3	MO/water 60:40	91		
		99		
			156	
A4	MO/bR buffer 60:40		149	49
A5	MO/bR buffer, crystallization buffer 50:50	91		
			147	

XFEL (vacuum)				
V1	MO/water 60:40	87.1	139.2	44.9
V2	MO/water 85:15			37.5, 49.3
V3	MO/bR buffer 60:40, 50 µm nozzle	95.1	150.7	45.2

Plate (control)				
C1	MO/water 60:40		144.5	
C2	MO/water 60:40		120.0	
C3	MO/water 60:40 (aged)	99.0	155.1	
C4	MO/water 60:40 (overmixed)	106.1		
